# Foreign Accent Syndrome Mimicked by Garcin Syndrome with Spontaneous Resolution

**DOI:** 10.1155/2008/498652

**Published:** 2008-12-18

**Authors:** Michael Hoffmann

**Affiliations:** Department of NeurologyUniversity of South FloridaTampaFLUSA

## Abstract

An English speaking women developed a French accent, without any aphasic syndromes, in conjunction with multiple left sided cranial nerve deficits, temporally related to cranial trauma. Extensive testing with multimodality magnetic resonance imaging, cerebrospinal fluid and laboratory analysis was unremarkable. She was followed over a 3 year period during which her French accent resolved as did the majority of her multiple unilateral cranial neuropathies. The neurological diagnoses included a foreign accent syndrome attributed to a reversible Garcin syndrome.

## 1. Index patient

A 63 year old woman, English speaking, born in the USA, presented with left facial paresis intermittent diplopia and alteration in her voice that her family consistently described as a French accent and corroborated by the author and witnessed by 2 other neurologists. This occurred temporally related to a whiplash injury incurred in a motor vehicle accident 3 weeks previously, noticed by the patient about one week after the incident. Relevant comorbid history included hypertension, diet controlled diabetes, benign essential tremor and dystonia. Examination revealed an alert and cooperative, normotensive, apyrexial woman with normal cognitive examination. This included a Minimental score of 30/30 and normal Boston Naming Test score of 53/60. Cranial nerve evaluation was notable for normal pupils, left ptosis, left eye abduction paresis left facial hypoesthesia (V1-3 distribution) and left facial weakness. The Rinne test was abnormal in that bone conduction was superior to air conduction bilaterally and the Weber test localized to the right. Pharyngeal and palatal sensation was intact but her uvula deviated to the right and the tongue deviated to left. The sensorimotor system was normal, without reflex loss or asymmetry noted. No incoordination or gait problem was apparent. In summary she had neurological deficits confined to the cranial nerves enumerated by left 3, 5, 6, 7, 8, 9, 10 and 12.

The differential diagnosis necessitated a work up to exclude extrinsic base of brain pathological processes such as infectious, inflammatory, metastatic and vascular disease, including brainstem strokes as well as other entities such as sarcoidosis, meningeal carcinomatosis, metastatic disease, mucormycosis, lymphoma, and primary tumours such as chemodectoma and nasopharyngeal cancer. From a clinical point of view, the findings were consistent with a Garcin syndrome because of the almost universally left sided affliction of the cranial nerves, either on the basis of post traumatic cranial nerve deficits or post viral etiology. The latter was corroborated by normal investigations that included a negative RPR, normal TSH, HbA1C, normal cerebrospinal fluid studies that were also negative for HSV 1 and 2, acid fast bacilli, culture negative, no yeast present and West Nile Virus negative. The opening pressure 10–12 cm of water and the cytospin negative for malignancy but abnormal rare lymphocytes noted. Autoimmune and chronic inflammatory and granulomatous disorders testing was likewise negative with C -anca and P-

ISSN 0953-4180/08/$17.00 © 2008 – IOS Press and the authors. All rights reserved anca negative, ANA negative, angiotensin converting enzyme level normal, liver function tests normal, B12, Folate normal with the lipid panel revealing minor elevations in total cholesterol but not LDL. Homocysteine and CRP were also within normal limits. Neuroradiological studies revealed three normal MRI parenchymal brain scans, including normal brainstem (T1, T2, DWI, FLAIR) with contrast normal on three separate occasions over a 2 year follow up period, normal base of skull XR and CT brain scan and a normal catheter 4 vessel cerebral angiogram with specific attention to vasculitis with the only abnormal finding a web like structure of proximal left subclavian. Cervical spine MR studies were likewise unremarkable apart from stenosis and moderate spondylosis C5–7. Sonographic studies including cervicocephalic duplex Doppler normal and echocardiography were unremarkable with an ejection fraction of 62% and 73% on two occasions. Chest radiography revealed no pathology and ECG rhythm was sinus. Follow up over a 3 year period documented an overall improvement in her condition most notably her speech which had recovered from the accent but with residual mild dysarthria with fluent speech and good comprehension.

## 2. Discussion

Garcin syndrome is a stepwise deterioration of the unilateral 12 cranial nerves, or the majority (some say 7 or more) first described in 1926 by Garcin and Guillain [3]. The causes invoke a work up for extramedullary multiple cranial nerves palsies that include meningeal processes (carcinomatous or lymphomatous meningitis), infectious radiculitis (Lyme disease, syphilis, tuberculosis, fungal infections, CMV, Herpes Zoster, HIV), ii) base of skull lesions such as metastases, nasopharyngeal tumour, sarcoma, chordoma, trauma, dissection of the carotid artery or jugular vein thrombosis, craniocervical junction abnormalities (basilar invagination, Arnold Chiari malformation), iii) perineural invasion by processes such as squamous or basal cell carcinoma, inflammatory and infectious processes such as Sarcoidosis, Wegener's granulomatosis, Guillain Barre syndrome and other viral parainfectious or post infectious syndromes, iv) autoimmune disease (mixed connective tissue disease and idiopathic v) Melkerson Rosenthal syndrome, Tolosa Hunt syndrome [8]. Although Garcin syndrome often connotes a sinister etiology, viral and post traumatic etiologies have been reported with complete recovery [7]. In this patient either trauma (temporal relationship) or a viral infection (by exclusion and presence of lymphocytes in CSF) such as brainstem encephalitis are the most likely explanations. Significant cerebrovascular stenoses, occlusions and other vasculopathies were excluded by 4 vessel catheter angiography.

Intriguing though in this patient was the development of a Foreign Accent Syndrome (FAS) due to an extra axial brain lesion, the first such described case [1, 4,5]. Foreign accent syndrome, a rare speech disorder whereby the person's pronunciation closely resembles that of an identified other accent by like speaking people, has been associated with motor aphasias secondary to vascular lesions and neurodegenerative disease [2, 9,10]. FAS is an important disorder to recognize as the patients may not have aphasia as such [10] but lesions often noted in the left Broca's region or subcortically. A number of accents of been reported that include, Brazilian to North American, English to French, Japanese to Korean, American to Irish. Interestingly the majority were described in English or American English patients with a change to a French accent [10]. An attractive current theory conjectures that impairment of the functional interplay between cerebral and cerebellar speech enters involved in motor speech planning results in FAS [6]. In a sense therefore, it is a disorder of the motor speech components of language which in this patient was due to motor cranial nerve impairment (combination of 7, 9, 10 and 12) rather than cortical network derived. This would be different to a mere dysarthria, a relatively common clinical presentation. It may be surmised that multiple cranial nerve involvement is required to develop this subcategory of FAS, in itself a rare occurrence. The etiology of the FAS mechanism is considered to be due to changes of rhythm, stress, intonation of articulation as well as the articulation of consonents, vowels and syllable structure. In this first ever described case of extra axial FAS, the message is to recognize both the speech syndrome as well as cranial neuropathy with its diverse differential diagnosis and be aware of the potentially good outcome.
